# Hematological and Biochemical Markers of Iron Status in a Male, Young, Physically Active Population

**DOI:** 10.1155/2014/349182

**Published:** 2014-06-22

**Authors:** Lázaro Alessandro Soares Nunes, Helena Zerlotti W. Grotto, René Brenzikofer, Denise Vaz Macedo

**Affiliations:** ^1^Faculty of Biomedical Sciences, Metrocamp College-IBMEC Group, 13035-270 Campinas, SP, Brazil; ^2^Department of Clinical Pathology, School of Medical Sciences, State University of Campinas, 13083-881 Campinas, SP, Brazil; ^3^Laboratory of Instrumentation for Biomechanics, Physical Education Institute, State University of Campinas, 13083-851 Campinas, SP, Brazil; ^4^Laboratory of Exercise Biochemistry-LABEX, Biochemistry Department, Biology Institute, State University of Campinas, 13083-970 Campinas, SP, Brazil

## Abstract

The aim of this study was to establish reference intervals (RIs) for the hemogram and iron status biomarkers in a physically active population. The study population included male volunteers (*n* = 150) with an average age of 19 ± 1 years who had participated in a regular and controlled exercise program for four months. Blood samples were collected to determine hematological parameters using a Sysmex XE-5000 analyzer (Sysmex, Kobe, Japan). Iron, total iron-binding capacity (TIBC), transferrin saturation and ferritin, and high-sensitivity C-reactive protein (CRP) concentrations in serum samples were measured using commercial kits (Roche Diagnostics, GmbH, Mannheim, Germany) and a Roche/Hitachi 902 analyzer. The RIs were established using the RefVal program 4.1b. The leucocyte count, TIBC, and CRP and ferritin concentrations exhibited higher RIs compared with those in a nonphysically active population. Thirty volunteers (outliers) were removed from the reference population due to blood abnormalities. Among the outliers, 46% exhibited higher CRP concentrations and lower concentrations of iron and reticulocyte hemoglobin compared with the nonphysically active population (*P* < 0.001). Our results showed that it is important to establish RIs for certain laboratory parameters in a physically active population, especially for tests related to the inflammatory response and iron metabolism.

## 1. Introduction

The physical training undertaken by athletes results in different degrees of microtrauma to the muscle. This microtrauma is related to the acute inflammatory response, which promotes muscle repair and regeneration. This response involves the production, recruitment, and delivery of proteins (e.g., cytokines, immunoglobulins, and acute phase proteins) and cells (e.g., leukocytes) in the circulation [1]. Acute and chronic exercise training produce different effects on hematological parameters. After a single bout of exercise, there is a rapid and pronounced neutrophilia due to demargination caused by shear stress and catecholamines, followed by a second delayed increase due to the cortisol-induced release of neutrophils from the bone marrow [2, 3]. Whereas the numbers of monocytes and lymphocytes can increase during and immediately after an exercise bout, the lymphocyte count falls below preexercise levels during the early stages of recovery, returning to basal levels within 4 hours [4]. All of these numbers generally return to basal levels within 3–24 hours [5].

Exercise training can influence immune function, health, and performance. In general, exercise training with low-to-moderate volume and intensity, with gradual increases, can enhance immune function and reduce the incidence of infections [3]. However, among highly trained and elite athletes, high-intensity training periods and strenuous physical exercise are associated with an increased susceptibility to upper respiratory tract infections (URTIs) [3, 6, 7]. Moreover, other factors, including lifestyle behaviors and nutritional status, can influence an athlete's immune function. Hence, monitoring an athlete's immune function through hematological parameters has become an important part of competition preparation [4].

Fully automated hematology analyzers have the capacity to quantify reticulocytes, the immature form of erythrocytes. The evaluation of immature red blood cell (RBC) parameters, including the number and hemoglobin content of reticulocytes, can be useful for monitoring positive adaptations to training or for identifying the use of prohibited substances to stimulate bone marrow production. Moreover, measuring hemoglobin concentration and reticulocyte parameters may be useful for diagnosing sports anemia, which can impair an athlete's performance. Persistent abnormalities in RBCs, hemoglobin concentration, and hematological indices can also indicate pathological conditions, such as deficits in iron, folic acid, or vitamin B12. Furthermore, other hematological abnormalities (thalassemia, sickle cell disease, and hereditary spherocytosis) can also alter an athlete's RBC profile [8, 9].

Athletic-induced iron deficiency is commonly detected in athletes, particularly those who engage in endurance sports. Iron is an essential component of hemoglobin, myoglobin, cytochromes, and other iron-containing proteins that participate in oxidative phosphorylation [10]. Additionally, macrophages can accumulate iron derived from RBCs, which is recycled by the reticuloendothelial system and thus participates in the immune defense against microbial pathogens [11]. In the bloodstream, iron is coupled to transferrin and can inhibit damage by reactive oxygen species (ROS) derived from Fenton's reaction [12]. In reticuloendothelial cells in the liver, spleen, and bone marrow, iron is stored as ferritin and hemosiderin [13].

To monitor immune function and iron status in athletes, it is important to understand the influence of exercise training on hematological and iron-related biochemical parameters. To increase the utility of these screening tests in physically active individuals, it is crucial to establish specific reference intervals in a physically active population, according to the International Federation of Clinical Chemistry (IFCC) rules. The aim of this study was to establish reference intervals for the hemogram, high-sensitivity C-reactive protein, and iron status biomarkers in young male individuals who had undergone 4 months of regular physical activity.

## 2. Materials and Methods

### 2.1. Participants

The study included five hundred (*n* = 150) healthy male volunteers with an average age of 19 ± 1 years. All the participants were in the first stage of physical and educational preparation for careers in the army. They were from different regions of the country, and, for one year, they had lived at the same place and had participated in the same numbers of hours of sleeping, eating, exercising, and studying. The volunteers participated in a regular and strictly controlled exercise program, which consisted predominantly of aerobic activities (high volume and different submaximal intensities), such as running and swimming. They had exercised three hours daily for four months in 2011 (from February to May). They had trained five days per week, with two days of rest. This group constituted a highly uniform group of young, physically active individuals. The participants provided written formal consent for participation in the research. The participants completed a questionnaire concerning their use of medication, complaints of pain, and injuries caused by training. Were selected for the reference group only those with no history of tobaccoism or chronic inflammatory disease. Those who were using medications, had not trained in the last three days, exhibited different clinical conditions (injuries related to training, muscle pain complaints, shin splint, or flu), or were suspected of congenital disorders (thalassemia or sickle cell disease) were analyzed separately as outliers. This study was approved by the University Ethics Committee for Research with Humans (CAAE: 0200.0.146.000-08). All the study procedures were in accordance with the Declaration of Helsinki.

### 2.2. Blood Sampling and Analysis

All blood samples were collected after two days of rest to avoid the effects of hemodynamic variations and acute hemodilution that are induced by exercise [14]. The blood samples were collected under the following standardized conditions: 2.0 mL of total venous blood was collected in vacuum tubes containing EDTA/K3 to determine hematological parameters, and 8.0 mL was collected in tubes with a Vacuette (Greiner Bio-one, Brazil) gel separator to obtain serum for biochemical measurements. The blood samples were collected in the morning after 12 hours of fasting, with the subjects being in a seated position. All the samples were then transported to the laboratory at 4°C and were analyzed within 60 min after the blood collection. The hematological parameters were obtained with a Sysmex XE-5000 automated analyzer, which uses a polymethine dye specific for RNA/DNA to facilitate reticulocyte enumeration and determinations of degree of immaturity and hemoglobin content. The e-Check (Lot 1144) Sysmex 3 levels were used as an internal quality control and were performed in parallel with the hematological tests. The means and standard deviation derived from the control samples were used to calculate the coefficient of analytical variation (CV_*A*_). The analyzed parameters and each respective CV_*A*_ were as follows: red blood cell count (RBC) (CV_*A*_ = 0.7%); blood hemoglobin concentration (Hb) (CV_*A*_ = 1.1%); hematocrit (Ht) (CV_*A*_ = 1.0%); mean corpuscular volume (MCV) (CV_*A*_ = 0.8%); erythrocyte distribution width (RDW) (CV_*A*_ = 0.9%); mean corpuscular hemoglobin (MCH) (CV_*A*_ = 1.0%); mean corpuscular hemoglobin concentration (MCHC) (CV_*A*_ = 1.2%); reticulocyte count (Ret) (CV_*A*_ = 3.7%); immature reticulocyte fraction (IRF) (CV_*A*_ = 10.0%); reticulocyte hemoglobin equivalent (Ret-He) (CV_*A*_ = 2.5%); white blood cell (WBC) (CV_*A*_ = 2.2%), lymphocyte (Lymph) (CV_*A*_ = 2.3%), neutrophil (Neut) (CV_*A*_ = 3.0%), monocyte (Mono) (CV_*A*_ = 6.2%), basophil (Baso) (CV_*A*_ = 2.5%), eosinophil (Eo) (CV_*A*_ = 7.5%); platelet (PLT) counts (CV_*A*_ = 2.6%); mean platelet volume (MPV) (CV_*A*_ = 1.0%); and immature platelet fraction (IPF) (CV_*A*_ = 5.6%). The biochemical measurements were conducted using commercial kits (Roche Diagnostics, GmbH, Mannheim, Germany) and a Roche/Hitachi 902 analyzer. Control serum was used to estimate the imprecision of the methods of biochemical analysis. The assays included the serum concentrations of iron (CV_*A*_ = 1.0%), ferritin (CV_*A*_ = 2.1%), and high-sensitivity C-reactive protein (h-CRP) (CV_*A*_ = 3.2%) as well as total iron-binding capacity (TIBC) (CV_*A*_ = 5.4%). The percent transferrin saturation (% TSAT) was calculated as follows: [iron/(TIBC)] × 100.

### 2.3. Statistical Analysis

The data were tested for Gaussian distribution using the Kolmogorov-Smirnov test. The Mann-Whitney test for nonparametric distribution was used to determine the differences between the high h-CRP (outliers) and normal h-CRP (reference individuals) groups. GraphPad Prism 6.0 for Mac OS X (GraphPad Software) was used to perform the statistical analyses and create the graphs. Values of *P* < 0.05 were considered significant. Reference intervals were established according to the IFCC rules using RefVal program 4.1 beta [15]. We calculated the nonparametric 2.5th and 97.5th percentiles, with their 90% confidence intervals (CIs), using a bootstrap methodology. Horn's algorithm was used to remove the outliers from the reference population [16].

## 3. Results

After the blood sample analyses, 22 individuals who exhibited abnormal blood results (leukocyte count > 12.0 × 10^9^/L, hemoglobin concentration < 120 g/L, or C-reactive protein level > 15.0 mg/L) [17, 18] or different clinical conditions (injuries related to training, muscle pain complaints, shin splint, or flu) were classified as outliers and were removed from the reference interval calculation. Additionally, eight individuals with mild microcytosis and higher RBC numbers, which are characteristics of thalassemia, were identified using the Mentzer index [19] and were excluded from the reference population and also included as outliers. As such, 120 healthy, physically active individuals constituted the reference sample group (Table 1).

Table 1 shows the reference intervals (2.5th and 97.5th percentiles) and the respective confidence intervals for the examined hematological parameters in male, young, physically active individuals after four months of regular and systematized training. The outliers detected by Horn's algorithm (indicated in Table 1) were removed from the reference interval calculation.

Table 2 shows the reference intervals and confidence intervals for iron status and acute phase proteins in male, young, physically active individuals.

**Figure 1:**
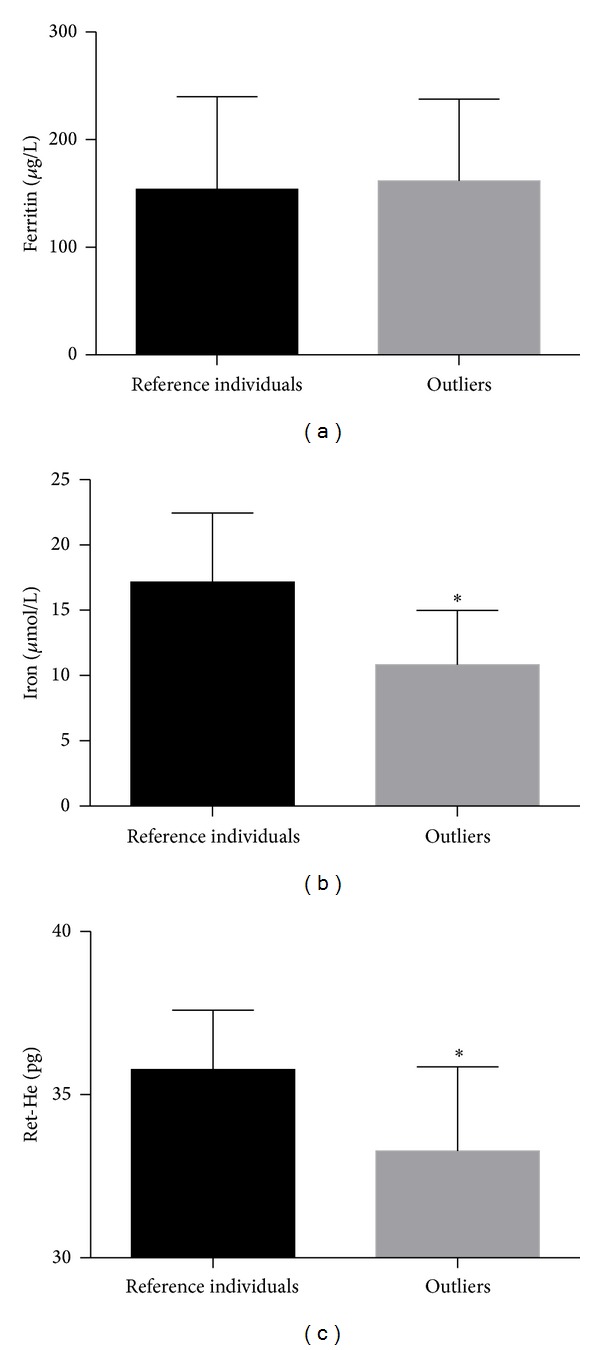
Ferritin (a), iron (b), and Ret-He (c) values in reference individuals (normal h-CRP) (*n* = 119) compared with outliers (higher h-CRP) (*n* = 14). The normal CRP reference range (<10.2 mg/L) was based on a reference population study. The graph shows the means ± standard deviation. *P < 0.001.

In this study, 30 subjects were classified as outliers due to abnormal blood results and were excluded from the reference interval calculation. Moreover, 46% (*n* = 14) of the subjects classified as outliers presented CRP values above the reference intervals for physically active subjects.  Figure 1 shows a comparison of the ferritin (a), iron (b), and Ret-He (c) values between the reference individuals (normal CRP concentrations) and outliers (higher CRP concentrations).

No differences in ferritin levels were observed between the normal and higher CRP groups (Figure 1(a)). The iron (Figure 1(b)) and Ret-He (Figure 1(c)) values were significantly lower in the higher CRP group compared with the reference group (*P* < 0.001).

## 4. Discussion

Previous studies from our group showed that different young, physically active individuals respond to four months of training stimulus similarly to athletes in terms of certain parameters in blood [17] and saliva [20], justifying the use of this young male trained population to establish reference intervals for sports medicine applications.

Exercise training appears to result in local and systemic imbalances in the anti-inflammatory, compared with the proinflammatory, status. This imbalance promotes tissue adaptation and protects the organism against the development of chronic inflammatory diseases and against the deleterious effects of overtraining, a condition in which systemic and chronic proinflammatory and prooxidant states appear to preponderate [21, 22]. In our population, we found slightly higher WBC and neutrophil counts in the lower percentile compared with healthy sedentary individuals (WBC = 3.5–9.8 × 10^9^/L and Neut = 1.5–7.0 × 10^9^/L) [23]. The 2.5th and 97.5th percentile monocyte counts (Table 1) were higher than those of a healthy, nonphysically active population (Mono = 0.2–0.64 × 10^9^/L) [23], indicating the effects of training on this parameter. Blood monocytes are the main source of tissue macrophages recruited in response to exercise [24], and they participate in the repair, growth, and regeneration of muscles [25].

There is little published evidence suggesting clinical differences between the immune functions of sedentary and physically active subjects in the true resting state (i.e., more than 24 h after the last training session) [1]. In our study of physically active subjects, we found a slightly higher lymphocyte count compared with a nonphysically active population (lymphocytes = 1.0–2.9 × 10^9^/L) [23].

The reference intervals for RBC parameters determined in our study did not differ from those in a nonphysically active population [23]. In sports medicine, mature and immature erythrocytes can be assessed to identify possible doping methods, such as the enhancement of oxygen transport by recombinant erythropoietin (rEPO) abuse. This indirect form of doping control was implemented by the World Anti-Doping Agency (WADA) through the hematological module of the athlete biological passport (ABP). As a part of the ABP, assessments of hemoglobin concentration and reticulocyte count allow the estimation of oxygen blood transport capacity and recent RBC production, respectively [26]. The reference intervals for reticulocyte count obtained in our study of physically active individuals were similar to those for a healthy nonphysically active population [23] and for athletes, including cyclists, swimmers, rugby players, tennis players, and soccer players [27–29].

The IRF in our population was relatively high compared with the general population (1.6–10.5%) using the same hematology analyzer [23]. The continuous stimulation of bone marrow due to accelerated iron metabolism, hypoxia, and exercise-induced hemolysis in athletes can partially explain this difference [8, 30]. Moreover, IRF can be a sensitive marker of erythropoietic activity in athletes undergoing altitude training or exposed to exogenous EPO stimuli [28].

Acute exercise bouts have been shown to promote an acute phase response, resulting in postexercise cytokine levels similar to those observed during sepsis or inflammatory disease. Strenuous exercise induces moderate increase in proinflammatory cytokines tumor necrosis factor *α* (TNF-*α*) and interleukin 1*β* [21, 31]. Interleukin-6 (IL-6) derived from the muscle cells can rise up to 100-fold during the exercise compared to preexercise levels [21]. CRP is the major acute phase protein associated with coronary events in apparently healthy subjects. Our CRP reference interval for physically active individuals (Table 2) was based on the 97.5th percentile. Other studies have found similar results for the 95th percentile in healthy, nonphysically active populations [32–34]. However, it is important to emphasize that these values are above the value (>3.0 mg/L) used to determine groups at high risk of cardiovascular disease, which is based on CRP [34]. Interleukin-6, which is produced in response to continuous training, can stimulate the synthesis of acute phase proteins by hepatocytes and can promote an elevated steady-state CRP concentration compared with healthy, nonphysically active subjects.

Iron, TIBC, and ferritin levels are traditional biomarkers for screening athletes during the training season [35]. Iron deficiency may have a negative impact on oxygen transport and immune defense, thus influencing athletic performance [36]. The main mechanisms of exercise-related iron loss in athletes include hemolysis, hematuria, sweating, gastrointestinal bleeding, and chronic inflammation [36]. Iron metabolism must be tightly regulated to supply iron as needed while avoiding the toxicity associated with iron excess. The main stimuli to regulate iron homeostasis are hypoxia, iron deficit, iron overload, and inflammation. In addition, iron metabolism is influenced by nutritional status, age, gender, bone marrow activity, and some pathological conditions such as bacterial infections [37]. Furthermore, recent reports have suggested that hepcidin, an iron-regulatory peptide hormone mainly produced in the liver, can regulate plasma iron concentrations in response to inflammation [38]. Hepcidin regulates iron concentration and tissue iron distribution via the inhibition of intestinal absorption, released by macrophages and iron mobilization from hepatic stores [39]. The primary mediator for the upregulation of hepcidin is IL-6 [40, 41]. Hepcidin levels increase 3–24 hours after exercise in response to IL-6, producing rapid decreases in the plasma iron concentration [40, 41]. The hepcidin synthesis decreases in iron deficiency, anaemia, and hypoxia [37]. The two last conditions are associated with increased erythropoiesis secondary to a rise of EPO secretion [42].

In our study, some subjects were classified as outliers due to high CRP concentrations and were excluded from the reference interval calculation. They had lower iron and hemoglobin reticulocyte concentrations (Ret-He), similar to those found in individuals with anemia of chronic disease (ACD). ACD results from the activation of the immune and inflammatory systems, resulting in the increased production of inflammatory cytokines that reduce the rate of iron mobilization from tissue stores [43]. The degree of hemoglobinization in reticulocytes is used to detect functional iron deficits and enables the early evaluation of bone marrow activity [44].

Ferritin levels, a biomarker of iron stores, did not differ between the higher and normal CRP groups (Figure 1(a)). Ferritin is an acute phase protein that is upregulated by tumor necrosis factor *α* (TNF-*α*) and interleukin 2 (IL-2), two proinflammatory cytokines produced during and after strenuous exercise [3, 45]. It is possible that the chronic inflammation state present in these subjects masked the changes in plasma ferritin levels. Although the results indicate a link between chronic inflammation and iron status parameters in these individuals, one limitation of this study was the lack of blood sample collection before the exercise program to identify the possibility of a prior inflammatory state. Thus, our comparisons were based on prior published studies conducted under specific conditions using different instruments. This limitation is particularly relevant for several parameters, such as monocytes, IRF, and reticulocytes [23]. Future investigations could verify this association in a larger sample including TNF-*α*, IL-2, and IL-6 results.

## 5. Conclusions

In conclusion, our results showed that it is important to consider exercise training when establishing reference intervals for several specific parameters, mainly those related to the inflammatory response. In addition, to avoid deficits in iron, an element that is crucial for an athlete's performance, it is important to monitor iron status using new hematological parameters and inflammatory biomarkers.

## Figures and Tables

**Table 1:** The reference intervals, confidence intervals, and outliers excluded by Horn's algorithm for hematological parameters in a male, young, physically active population.

Analyses	Reference interval	90% confidence interval		Subjects (*n*)	Outliers (*n*)
	2.5th–97.5th	2.5th	97.5th		
WBC (10^9^/L)	5.0–10.8	4.9–5.2	10.4–11.7	119	1
Lymph (%)	15.0–48.0	14.0–19.0	43–53	119	1
Lymph (10^9^/L)	1.3–3.7	1.2–1.4	3.3–4.1	119	1
Neut (%)	37.0–72.0	33.0- 41.0	67.0–76.0	120	—
Neut (10^9^/L)	2.4–7.5	2.3–2.5	6.6–8.0	119	1
Mono (%)	7. 0–13.0	6.5–7.5	13.0–15.0	118	2
Mono (10^9^/L)	0.4–1.4	0.3–0.5	1.1–1.5	118	2
Eo (%)	0.9–7.7	0.8–1.2	7.0–7.8	114	6
Eo (10^9^/L)	0.05–0.55	0.04–0.08	0.53–0.87	118	2
Baso (%)	0.4–1.8	0.3–0.4	1.5–1.9	117	3
Baso (10^9^/L)	0.02–0.11	0.01–0.03	0.11–0.15	120	—
PLT (10^9^/L)	141–305	133–153	284–320	120	—
MPV (fL)	9.8–13.4	9.7–10.1	13.0–13.7	120	—
IPF (%)	2.0–10.4	1.7–2.2	9.9–11.8	119	1
RBC (10^12^/L)	4.77–5.72	4.74–4.84	5.57–5.77	118	2
Ht (%)	40.6–47.4	39.4–41.0	46.6–48.9	120	—
Hb (g/L)	133–162	132–135	158–163	118	2
MCV (fL)	80.0–89.5	79.5–80.7	88.6–91.2	118	2
MCH (pg)	26–30.0	24–26	30-31	120	—
MCHC (g/L)	32–35	31–32	34–35	119	1
RDW (%)	12.4–14.9	12.4–12.7	14.7–15.2	119	1
RDW-SD (fL)	38–45	37–38	44–46	120	—
RET (%)	0.54–1.33	0.5-0.6	1.2–1.4	120	—
RET (10^9^/L)	28–68	24–30	64–75	120	—
IRF (%)	3–14	2.7–3.6	11.5–15.0	119	1
Ret-He (pg)	32.2–39.2	31.6–33.0	38.5–39.4	119	1

**Table 2:** The reference intervals, confidence intervals, and outliers excluded by Horn's algorithm for biochemical parameters in a male, young, physically active population.

Analyses	Reference interval	90% confidence interval		Subjects (*n*)	Outliers (*n*)
	2.5th–97.5th	2.5th	97.5th		
h-CRP (mg/L)	0.2–10.2	0.2-0.3	7.0–14.8	120	—
Iron (*μ*mol/L)	8.4–28.8	8.2–8.9	27.5–31.6	119	1
TIBC (*μ*mol/L)	43.8–64.5	43.1–45.0	63.1–68.9	120	—
%TSAT	19–45	19-20	43–46	119	1
Ferritin (*μ*g/L)	47–331	41–53	264–436	119	1
